# Supplementary material for: Preparation of Hot-Melt Extruded Dosage Form for Enhancing Drugs Absorption Based on Computational Simulation
Source: Pharmaceutics. 2020 Aug 11;12(8):757. doi: 10.3390/pharmaceutics12080757 (PMC7463902; doi:10.3390/pharmaceutics12080757)
Supplement: Supplementary file 1 [file pharmaceutics-12-00757-s001.pdf]

pharmaceutics

Preparation of Hot-Melt Extruded Dosage Form for Enhancing Drugs Absorption Based on Computational Simulation

Sung-Min Choi, Sung-Hoon Lee, Chin-Yang Kang and Jun-Bom Park

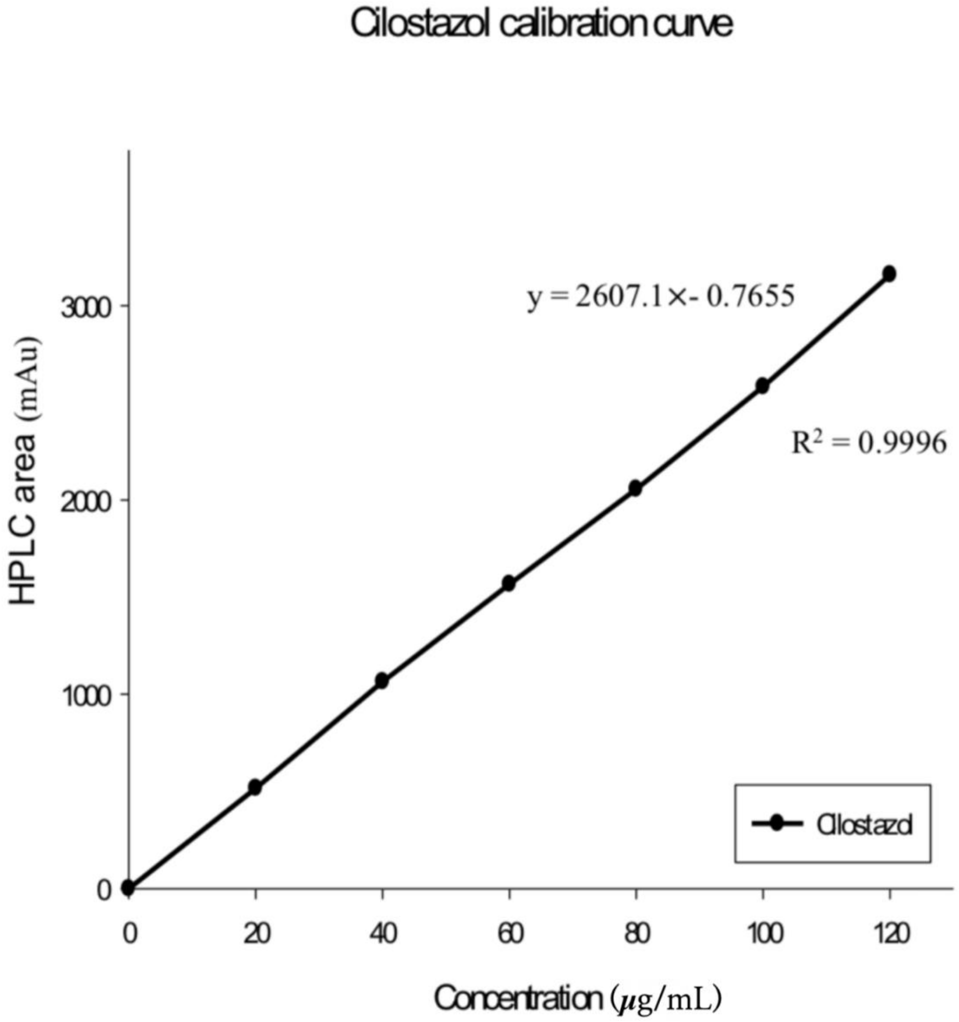

Figure S1. The linearity of the calibration curve with HPLC method, ranging from 20 μg/mL to 120 μg/mL.
